# Comparative Analyses of Vertebrate Gut Microbiomes Reveal Convergence between Birds and Bats

**DOI:** 10.1128/mBio.02901-19

**Published:** 2020-01-07

**Authors:** Se Jin Song, Jon G. Sanders, Frédéric Delsuc, Jessica Metcalf, Katherine Amato, Michael W. Taylor, Florent Mazel, Holly L. Lutz, Kevin Winker, Gary R. Graves, Gregory Humphrey, Jack A. Gilbert, Shannon J. Hackett, Kevin P. White, Heather R. Skeen, Sarah M. Kurtis, Jack Withrow, Thomas Braile, Matthew Miller, Kevin G. McCracken, James M. Maley, Vanessa O. Ezenwa, Allison Williams, Jessica M. Blanton, Valerie J. McKenzie, Rob Knight

**Affiliations:** a Department of Pediatrics, School of Medicine, University of California San Diego, La Jolla, California, USA; b Institut des Sciences de l’Evolution de Montpellier (ISEM), CNRS, EPHE, IRD, Université de Montpellier, Montpellier, France; c Department of Animal Sciences, Colorado State University, Fort Collins, Colorado, USA; d Department of Anthropology, Northwestern University, Evanston, Illinois, USA; e School of Biological Sciences, University of Auckland, Auckland, New Zealand; f Department of Botany, Biodiversity Research Centre, University of British Columbia, Vancouver, British Columbia, Canada; g Integrative Research Center, Field Museum of Natural History, Chicago, Illinois, USA; h University of Alaska Museum, Fairbanks, Alaska, USA; i Department of Vertebrate Zoology, National Museum of Natural History, Smithsonian Institution, Washington, DC, USA; j Center for Macroecology, Evolution and Climate National Museum of Denmark, University of Copenhagen, Copenhagen, Denmark; k Institute for Genomics and Systems Biology, University of Chicago, Chicago, Illinois, USA; l Committee on Evolutionary Biology, University of Chicago, Chicago, Illinois, USA; m Department of Biology, University of Florida, Gainesville, Florida, USA; n Sam Noble Oklahoma Museum of Natural History, Department of Biology, University of Oklahoma, Norman, Oklahoma, USA; o Department of Biology, University of Miami, Coral Gables, Florida, USA; p Rosenstiel School of Marine and Atmospheric Sciences, University of Miami, Miami, Florida, USA; q Human Genetics and Genomics, Hussman Institute for Human Genomics, University of Miami Miller School of Medicine, Miami, Florida, USA; r Institute of Arctic Biology, University of Alaska, Fairbanks, Fairbanks, Alaska, USA; s Moore Laboratory of Zoology, Occidental College, Los Angeles, California, USA; t Odum School of Ecology, University of Georgia, Athens, Georgia, USA; u Department of Infectious Diseases, College of Veterinary Medicine, University of Georgia, Athens, Georgia, USA; v Marine Biology Research Division, Scripps Institution of Oceanography, University of California San Diego, La Jolla, California, USA; w Department of Ecology and Evolutionary Biology, University of Colorado Boulder, Boulder, Colorado, USA; x Department of Computer Science & Engineering, Jacobs School of Engineering, University of California San Diego, La Jolla, California, USA; y Center for Microbiome Innovation, University of California San Diego, La Jolla, California, USA; University of Connecticut

**Keywords:** diet, evolution, flight, microbiome, vertebrate

## Abstract

Diet and host phylogeny drive the taxonomic and functional contents of the gut microbiome in mammals, yet it is unknown whether these patterns hold across all vertebrate lineages. Here, we assessed gut microbiomes from ∼900 vertebrate species, including 315 mammals and 491 birds, assessing contributions of diet, phylogeny, and physiology to structuring gut microbiomes. In most nonflying mammals, strong correlations exist between microbial community similarity, host diet, and host phylogenetic distance up to the host order level. In birds, by contrast, gut microbiomes are only very weakly correlated to diet or host phylogeny. Furthermore, while most microbes resident in mammalian guts are present in only a restricted taxonomic range of hosts, most microbes recovered from birds show little evidence of host specificity. Notably, among the mammals, bats host especially bird-like gut microbiomes, with little evidence for correlation to host diet or phylogeny. This suggests that host-gut microbiome phylosymbiosis depends on factors convergently absent in birds and bats, potentially associated with physiological adaptations to flight. Our findings expose major variations in the behavior of these important symbioses in endothermic vertebrates and may signal fundamental evolutionary shifts in the cost/benefit framework of the gut microbiome.

## Abstract (precis)

In this comprehensive survey of microbiomes of >900 species, including 315 mammals and 491 birds, we find a striking convergence of the microbiomes of birds and animals that fly. In nonflying mammals, diet and short-term evolutionary relatedness drive the microbiome, and many microbial species are specific to a particular kind of mammal, but flying mammals and birds break this pattern with many microbes shared across different species, with little correlation either with diet or with relatedness of the hosts. This finding suggests that adaptation to flight breaks long-held relationships between hosts and their microbes.

## INTRODUCTION

The mammalian gut microbiome has emerged as a key regulator of host physiology (1), and coevolution between host and microbial lineages has played a key role in the adaptation of mammals to their diverse lifestyles. Diet, especially herbivory, is an important correlate of microbial diversity in mammals (2, 3). Most mammalian microbiomes are also strongly correlated with host phylogeny, despite profound shifts in diet (2, 4–6). This suggests host factors that themselves change across host phylogeny, such as gut physiology, play an important role in structuring the gut microbiomes across mammals. The vertebrate adaptive immune system is even speculated to have evolved as just such a factor for selective maintenance of symbiotic homeostasis (7).

The importance of phylogeny-correlated factors to the diversity of vertebrate microbiomes more generally is still poorly understood. Phylosymbiosis, or the observation that more closely related host species have more similar microbiomes (8, 9), has been described in a number of nonmammalian taxa (10, 11). Other analyses have found substantial variation in phylosymbiotic signals among mammalian taxa (12), sometimes with conflicting results (13, 14). The presence of a robust phylosymbiotic correlation implies that host factors control microbial assembly. Even if the specific mechanisms are unknown, variation in the strength or presence of a measurable phylosymbiotic signal across host phylogeny could prove useful for identifying such mechanisms through comparative studies. However, most studies to date have focused on just a few taxa at a time, and variable methods for both surveying the microbiome and measuring phylosymbiosis and host specificity (or the restriction of microbes to specific host lineages) have made generalizations difficult.

Without broader evolutionary context, it is unclear how universally conserved patterns of host-microbe phylosymbiosis actually are. Growing evidence indicates that the strong patterns identified in mammals are the exception rather than the rule in vertebrates. Meta-analyses of fish (15) and birds (16) have failed to detect the strength of correlations to diet and phylogeny reported in mammals. A recent analysis of samples from more than 100 vertebrate species also found the strength of phylogenetic correlation to be much higher in mammals than in birds, reptiles, amphibians, or fish (17). It is increasingly appreciated in nonvertebrate animals that fundamental aspects of the host’s relationship to its symbiotic community can change drastically between taxa: many insects depend entirely on microbes for key metabolites, while others seem to be devoid of resident gut microbes (18). The complexity of different factors likely influencing diverse vertebrate gut communities remains a challenge to uncovering the most important causal relationships. The diversity of animal physiologies, habitats, and lifestyles offers opportunities to use convergent “natural experiments” in evolutionary history to sort through this complexity. By filling out our knowledge of microbial diversity across the vertebrate tree of life, we may be able to use major transitions in the host-microbiome relationship to identify elements of animal biology that are most likely to play such causal roles.

**FIG 1:**
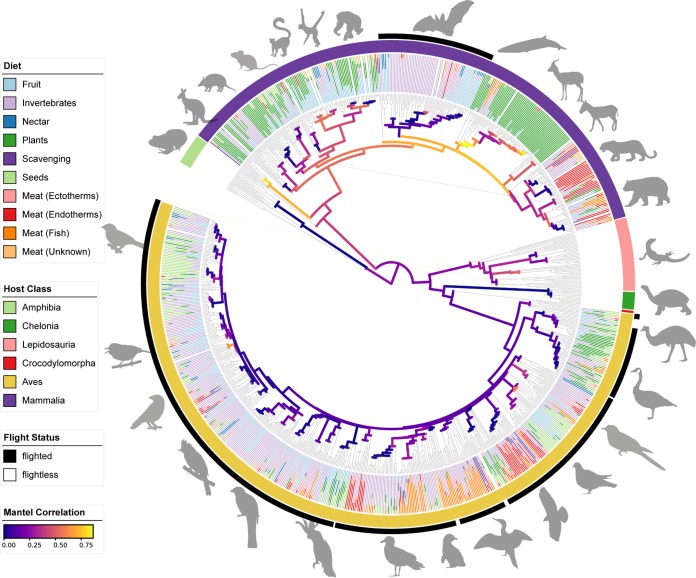
Host tree with diet composition as a bar chart, host taxonomic class in the inner ring, flight status as the outer ring, and strength of phylosymbiosis (Mantel Pearson correlation) plotted as branch color.

Reasoning that convergences in host phenotypes offer some of the clearest such natural experiments, we have assembled a large sample set using the Earth Microbiome Project standard methods (19) to identify patterns of phylosymbiosis and host specificity across vertebrates. Subsets of these data have previously explored convergences in diet, including convergent evolution of myrmecophagy in mammals (20), folivory in primates (4), and blood feeding in birds and mammals (21), as well as the impacts of captivity on the gut microbiome (22). Here, we present our first analysis of the complete data set, spanning samples from 315 mammalian species, 491 avian species, and 86 species representing other vertebrate classes (Fig. 1; see Data Set S1 in the supplemental material). Through large-scale vertebrate host sampling using consistent methods, we are able to compare broad patterns in the diversification of microbiomes both within and between major vertebrate lineages with substantially greater power.

10.1128/mBio.02901-19.1 DATA SET S1 Sample metadata. Download Data Set S1, XLSX file, 2.2 MB. Copyright © 2019 Song et al. 2019 Song et al. This content is distributed under the terms of the Creative Commons Attribution 4.0 International license.

## RESULTS

**FIG 2:**
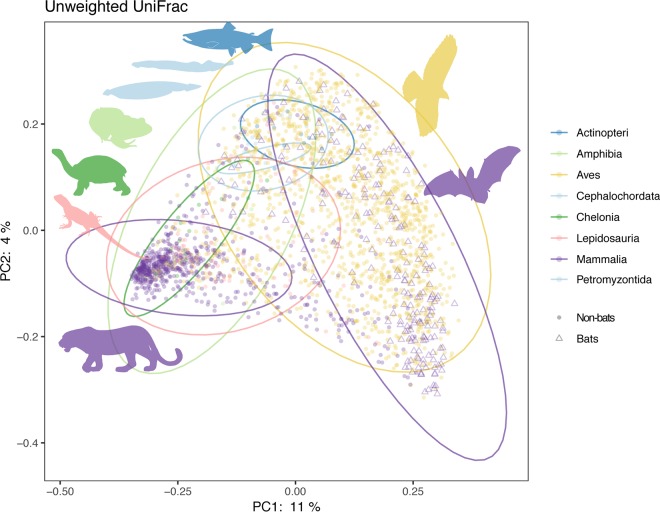
Principal-coordinate analysis of unweighted UniFrac distances between samples rarefied to 10,000 sequences/sample and filtered to include only up to 5 individuals per species (2,258 points). Colors represent host class, with the mammalian order Chiroptera shown as triangles; 95% confidence intervals per class represented by colored ellipses (separately for bats and nonflying mammals; Crocodylomorpha [crocodilians] excluded due to low sample number).

Principal-coordinate analysis (PCoA) of the complete microbiome data set reveals a striking pattern: although microbiomes from each vertebrate class generally cluster in PCoA space (permutational multivariate analysis of variance [PERMANOVA] *R*^2^ = 0.04897, *P* = 0.001), bats group more closely with birds than with other mammals or any other vertebrate class (Fig. 2). Furthermore, both of these clades harbor relatively low proportions of *Bacteroidetes* but high proportions of *Proteobacteria* (see Fig. S1C and D in the supplemental material), a phylum of bacteria highly associated with birds (16, 23) and flight (Fig. S1A and B) that is diminished in most nonbat mammalian hosts. Yet, despite these compositional similarities in the gut microbiomes of birds and bats, we find that the strongest similarity between these clades is a lack of strong association with a specific microbiome (Fig. 1).

10.1128/mBio.02901-19.4 FIG S1 Ranks of the log-ratio abundance changes of microbial phyla in mammals compared to that in birds and flighted mammals and birds compared to flightless mammals and birds. The *y* axes represent the log-fold change that is known up to some bias constant K, and the *x* axes numerically order the ranks of each phylum. (A) Phyla with positive values are those associated with birds, while those with negative values are associated with mammals. (B) Phyla with positive values are those associated with flightlessness, while those with negative values are associated with flight. Of the phyla showing high prevalence in the dataset (>90%), *Proteobacteria* rank among those highly associated with birds and/or with flight, while *Bacteroidetes* rank among those highly associated with flightlessness. Comparisons of relative abundances of *Proteobacteria* and *Bacteroidetes* across mammalian and bird orders, ordered by increasing median. (C) Bats (Chiroptera) have the highest levels of *Proteobacteria*, a phylum that is found in relatively low abundance in other mammals but common to most avian gut samples. Note that the smallest amounts of *Proteobacteria* are in ostriches (Struthioniformes), a flightless bird. (D) In contrast, bats have among the lowest levels of *Bacteroidetes*, a phylum that is found in relatively high abundance in other mammals and also less common to most avian gut samples. Download FIG S1, PDF file, 1.1 MB. Copyright © 2019 Song et al. 2019 Song et al. This content is distributed under the terms of the Creative Commons Attribution 4.0 International license.

In birds, we find gut microbiota in general to be only weakly correlated with host phylogeny (multiple regression on distance matrices [MRM] *R*^2^ = 0.02, *P* = 0.001) (Fig. S2B; Table S1) and not correlated with host diet (*P* = 0.415) (Fig. S3A) despite diet varying widely among bird lineages; this is juxtaposed with mammals, in which we see a strong correlation to both diet and phylogeny (*R*^2^ = 0.17, *P* = 0.001) (Fig. S2A to S3B and Table S1). Rather, correlations between bird gut microbiota and host phylogeny are more similar to levels in the less extensively sampled nonavian reptiles (*R*^2^ = 0.03, *P* = 0.002) and amphibians (*R*^2^ = 0.03, *P* = 0.021). An analysis of beta diversity through time (BDTT), a technique previously shown to deconvolute the contributions of host diet and phylogeny to microbiome diversity in mammals (5), also showed little correlation between bird diet and phylogeny at any depth of the microbial tree (Fig. S4C). Similarly, bats had lower correlations between gut microbiota and both diet and phylogeny than other well-sampled mammalian lineages in a BDTT analysis. Correlations between gut alpha diversity and host body mass also held much more weakly for all birds (*P* = 0.0101) than mammals (*P* < 0.001) (Fig. S5C), but only held for flightless mammals when considering bats and flightless birds separately (*P*_birds, flighted_ = 0.08, *P*_birds, flightless_ = 0.69, *P*_mammals, flighted_ = 0.07, *P*_mammals, flightless_ < 0.001) (Fig. S5D).

10.1128/mBio.02901-19.2 TABLE S1 MRM regression effect sizes of diet and phylogenetic distance. Download Table S1, DOCX file, 0.1 MB. Copyright © 2019 Song et al. 2019 Song et al. This content is distributed under the terms of the Creative Commons Attribution 4.0 International license.

10.1128/mBio.02901-19.5 FIG S2 Principal-coordinate ordinations of unweighted UniFrac distances, colored by taxonomic order of host. (A) Mammals. Bats indicated by open triangles, all other mammals by closed circles. (B) Birds. Flightless birds indicated by open triangles, all other birds by closed circles. Download FIG S2, PDF file, 1.0 MB. Copyright © 2019 Song et al. 2019 Song et al. This content is distributed under the terms of the Creative Commons Attribution 4.0 International license.

10.1128/mBio.02901-19.6 FIG S3 Principal-coordinate ordinations of unweighted UniFrac distances for bird microbiomes (A) and mammal microbiomes (B), colored by dietary compositions of host. Principal coordinate axes 1 and 2 are shown. Diet data derived from the EltonTraits ecological database (49) and represent 0% to 100% compositional scores across several categories. Here, we have combined the EltonTraits values for vertebrate prey (Diet.Vend [endotherms], Diet.Vect [ectotherms], Diet.Vfish [fish], Diet.Vunk [unknown]) into a single category “Diet.Meat.” Points are colored such that brighter colors indicate a higher dietary proportion for that category in each subplot. Download FIG S3, PDF file, 2.5 MB. Copyright © 2019 Song et al. 2019 Song et al. This content is distributed under the terms of the Creative Commons Attribution 4.0 International license.

10.1128/mBio.02901-19.7 FIG S4 Correlation between microbial community distance by unweighted UniFrac (A) and Jaccard (B) and host phylogenetic distance (estimated divergence time) for bird and mammal samples, restricted to within-class comparisons below 150 Ma. Points are fit with a generalized additive model in ggplot2 to capture nonlinear variation. (C) Amounts of variation in microbial beta diversity explained by host phylogeny and diet vary among host clades. The panel presents the correlation between microbial dissimilarities (as measured by the Jaccard index) and host phylogenetic or diet distances (*y* axis), plotted against the microbial phylogenetic scale at which microbial dissimilarities are measured (*x* axis; shallow phylogenetic resolutions [i.e., ASVs] on the left to deep phylogenetic resolutions on the right). Mammals show large amounts of association with diet in particular, across all microbial phylogenetic scales, while birds (Aves) show very low levels, and bats (Chiroptera) only show moderate levels at very shallow microbial phylogenetic scales. Download FIG S4, PDF file, 2.4 MB. Copyright © 2019 Song et al. 2019 Song et al. This content is distributed under the terms of the Creative Commons Attribution 4.0 International license.

10.1128/mBio.02901-19.8 FIG S5 (A) Microbiome alpha diversity in flighted and nonflighted birds and mammals. (Left) Faith’s phylogenetic diversity. All pairwise comparisons had a *P* value of <0.001, except for flighted versus flightless birds (*P* = 0.197). (Center) Shannon diversity. All pairwise comparisons had a *P* value of <0.001, except for flighted versus flightless birds (*P* = 0.024). (Right) Observed OTUs. All pairwise comparisons had a *P* value of <0.001, except for flighted versus flightless birds (*P* = 0.045). (B) Microbiome alpha diversity values for nonmigratory, partially migratory, and migratory birds. Microbiomes from migratory birds do not have significantly different measures of alpha diversity from nonmigratory birds for any metric (*P*_PD_ = 0.101, *P*_Shannon_ = 0.840, *P*_observed OTUs_ = 0.226). (C) Microbial alpha diversity scaling values for birds and mammals. (D) In nonflighted mammals, the amount of microbial alpha diversity scales with host body mass (*R*^2^ = 0.153, *P* < 0.001) as predicted by macroecological species-area relationships, except in bats, where the relationship appears to be inverse (*R*^2^ = 0.021, *P* = 0.040). In flighted birds, diversity and body mass are very marginally correlated (*R*^2^ = 0.003, *P* = 0.037), except in flightless birds (*R*^2^ = 0.014, *P* = 0.441). Download FIG S5, PDF file, 2.3 MB. Copyright © 2019 Song et al. 2019 Song et al. This content is distributed under the terms of the Creative Commons Attribution 4.0 International license.

**FIG 3:**
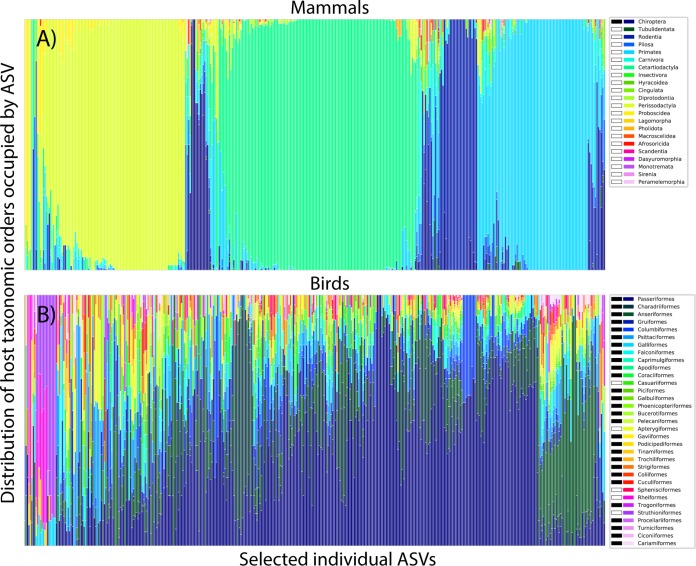
Taxonomic distributions of 400 randomly selected ASVs in mammals (A) and birds (B). Each column of the stacked bar chart corresponds to a different ASV. Different colored bars correspond to the taxonomic orders of each host sample in which that ASV is found, such that an ASV found in ten samples from the same host order would have a single colored column, while an ASV found in five samples each from two host orders would have a column evenly split into two colors. Note that on average, ASVs in mammals were only found in samples from a single mammalian order, while ASVs in birds were generally found in samples spanning many bird orders. ASVs with pink/purple bars on the left-most portion of panel B were found primarily in the flightless hosts of the Struthioniformes and Rheiformes orders (ostriches and rheas) within the Palaeognathae. Host orders capable of powered flight indicated by black bar in legend.

**FIG 4:**
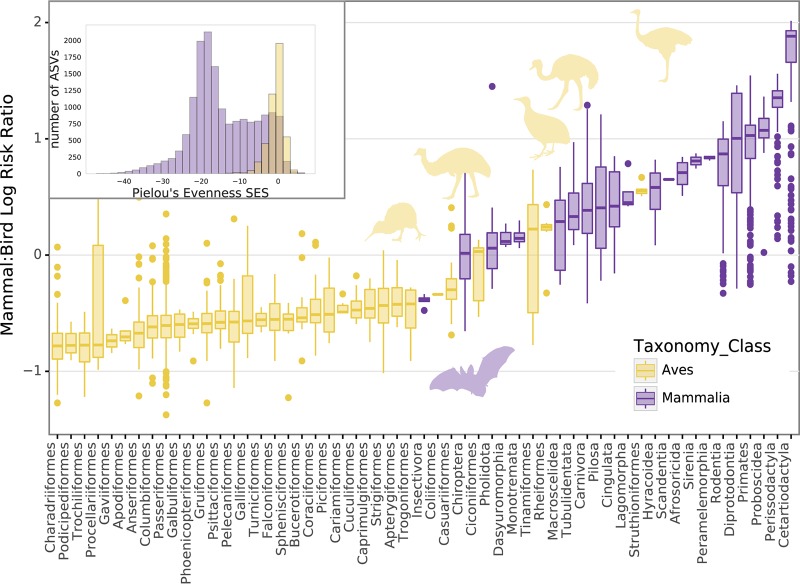
Flightless birds and bats have among the least bird- and mammal-specific microbiomes. Boxplot shows, per host sample grouped by taxonomic order, the mean log of the ratio of prevalence of an amplicon sequence variant (ASV) in mammals to its prevalence in birds. Samples from Chiroptera (bats) and palaeognath birds (Apterygiformes, Casuariiformes, Tinamiformes, Rheiformes, and Struthioniformes) are highlighted with silhouettes. (Inset) Distribution of the standardized effect size of Pielou’s evenness for ASVs across host orders from mammals and birds (see also Fig. 3). Across mammals, most ASVs show uneven distribution (high specificity), while across birds, nearly all ASVs show even or near even distribution.

The lack of correlation between host factors and microbial composition in birds appears to be partly a consequence of an overall lower level of specificity between microbial and host taxa than is found in mammals. While in mammals, most amplicon sequence variants (ASVs) are only found in a single host order, the opposite is true in birds: most ASVs are shared broadly across host taxa (Fig. 3). Using a standardized effect size measurement of Pielou’s evenness statistic to control for imbalances in our sampling, we find that microbial communities associated with mammals are generally much less evenly distributed across host taxa than are the microbial communities of birds (Fig. 4, inset). In particular, bats and other mammals from insectivorous and carnivorous orders harbored the least specific communities; while among the birds, the flightless orders (ostriches, emus, cassowaries, kiwis, and rheas), and the weak fliers from the related Tinamiformes (tinamous) were among those hosting communities with median specificities approaching those of mammals. The microbiota of these bird orders (constituting the Palaeognathae) were also more likely to occur in mammals, with samples from the Struthioniformes (ostriches), Rheiformes (rheas), and Tinamiformes hosting the least bird-specific microbiota, and samples from the Eulipotyphla (insectivorous shrews and moles), Dasyuromorphia (carnivorous marsupials), Pholidota (ant-eating pangolins), and Chiroptera (bats) hosting the least mammal-specific microbiota (Fig. 4).

**FIG 5:**
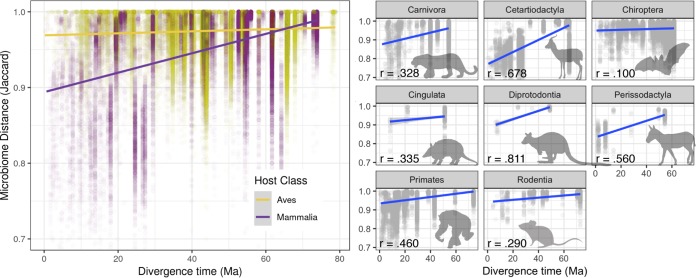
Mammals and birds show different patterns of phylosymbiosis. (Left) Within host orders, mammals show a generally strong correlation between host distance (estimated divergence time) and microbial community distance (Jaccard), but birds do not. (Right) Within mammalian orders with large sample size, bats (Chiroptera) show the weakest level of phylosymbiosis. For plots per bird order, see Fig. S7 in the supplemental material.

The association between flight and the loss of microbiome specificity is also reflected in measurements of phylosymbiosis, suggesting that the evolution of powered flight profoundly disrupts the pattern of phylosymbiosis between host and gut microbiome. We compared measurements of microbiome dissimilarity to host phylogenetic distance (measured as branch length on the TimeTree) (24) and approximate time-calibrated host phylogeny using the Mantel test (Fig. 1) (12, 25). We see that in mammals as a whole, the correlation between microbial community similarity and host phylogenetic distance is high, especially at recent timescales (Fig. S4A and B), whereas this correlation is much lower in birds (mammals: *P* = 0.001, *r* = 0.40, coefficient = 2.61E−4; birds: *P* = 0.001, *r* = 0.14, coefficient = 8.92E−5) (Fig. 5, left). In contrast, among mammalian orders, the strength of this correlation varies dramatically, with the steepest relationships between phylogenetic distance and microbiome turnover in the fermentative herbivores; among the well-sampled mammalian orders (≥7 species represented), bats were unique in having little correlation at all (Fig. 5, right).

10.1128/mBio.02901-19.10 FIG S7 Correlation between microbial community distance (Jaccard) and host phylogenetic distance (estimated divergence time) for bird orders with larger sample sizes (*n* ≥ 7). Download FIG S7, PDF file, 0.7 MB. Copyright © 2019 Song et al. 2019 Song et al. This content is distributed under the terms of the Creative Commons Attribution 4.0 International license.

## DISCUSSION

Our results suggest that the evolution of powered flight affected interactions between hosts and microbes in birds and bats convergently: birds and mammals with powered flight both display markedly less specificity than most nonflying mammals and somewhat less specificity than nonflying birds. We considered several potential proximate explanations for the convergent loss of microbial specificity in birds and bats, which can be broken down into ecological, evolutionary, and technical factors.

First, flight could expose individuals to more diverse-source microbial communities, resulting in increased microbial gut diversity if assembly of these communities was a neutral process. This seems unlikely to be an important factor in itself, as vertebrate gut communities have mostly been shown to follow nonneutral dynamics (26). If this were the case, however, we might expect both birds and bats to host especially high alpha diversity, reflecting colonization from a broader range of environments. Yet, the opposite appears to be the case (see Fig. S5A in the supplemental material): both birds and bats have lower mean alpha diversities than nonbat mammals (pairwise *t* tests, *P* < 0.001 for observed operational taxonomic units [OTUs], Shannon diversity, and Faith’s phylogenetic diversity [PD]). Furthermore, we did not see greater alpha diversity in migratory birds, which presumably encounter a greater range of environments than nonmigrators (*P* = 0.225 for observed OTUs) (Fig. S5B).

Similarities in microbiome composition and patterns of association suggest the presence of flight-correlated host factors exerting similar selective pressures on the assembly of the gut microbiome. Birds and bats both tend to have reduced intestinal lengths and shorter intestinal content retention times, perhaps as a by-product of selective pressure to decrease mass for more efficient powered flight (27–29). Shorter guts, with correspondingly reduced anaerobic volumes, could also present less of a barrier to microbial exchange through an aerobic environment. Accordingly, relative to that in nonflying mammals, both birds and bats have fewer obligate anaerobes (*P* < 0.001) and more facultative anaerobes (*P* < 0.001), and flightless birds have more obligate anaerobes than flighted birds (*P* = 0.001) as a proportion of their gut microbial communities, as measured by predicted high-level phenotypes using BugBase (30) (Fig. S6).

10.1128/mBio.02901-19.9 FIG S6 Microbial phenotype compositions for bird and mammal microbiomes inferred from BugBase (30). Flightless birds have higher predicted compositions of strictly anaerobic microbes than flighted birds (*P* = 0.001) and similarly for flightless mammals compared to bats (*P* < 0.001). Bats have significantly higher predicted composition of potential pathogens (*P* < 0.001). Download FIG S6, PDF file, 0.1 MB. Copyright © 2019 Song et al. 2019 Song et al. This content is distributed under the terms of the Creative Commons Attribution 4.0 International license.

It is possible that some other aspect of adaptation to flight has had a similar net effect on microbiome specificity. The metabolic demands of powered flight are profound and appear to have led to extensive and surprising degrees of convergence. Birds have the smallest genomes of all amniotes, and birds and bats both have more compact genomes than their flightless relatives, most likely due to extensive DNA loss—hypothesized to be related to powerful ongoing selection to reduce mass and enhance flight-related correlates of metabolism (31, 32). It is therefore plausible that pressure to decrease mass may extend to microbial biomass. Both birds and bats also have greater rates of intestinal paracellular absorption than nonflying vertebrates (28, 29), meaning that a higher proportion of simple nutrients are absorbed directly by the host, potentially decreasing the role for symbiotic microbial metabolism. Indeed, some studies of microbial biomass across animal hosts have reported that both birds and bats carry much lower numbers of microbial cells in feces than nonflying mammals (33). A selection toward microbial reduction may also explain in part why birds and bats both seem to have lost an association with *Bacteroidetes* but retained an association with *Proteobacteria*, a group of bacteria suggested to have high functional variability (34): to maximize microbial function while also reducing diversity and mass. Even if selective pressure to reduce overall microbial biomass did not, in itself, lead to the loss of tight evolutionary ties with specific symbionts, consistently lower resident microbial biomass might increase the proportion of transient environmental microbes relative to the total pool of microbes in the sample, decreasing the observed degree of host specificity on average. Indeed, this could also help explain the dominance of *Proteobacteria*, which make up a large proportion of the airborne microbiome (35), a source to which flighted animals are constantly exposed.

If the convergent loss of microbial specificity in birds and bats is ultimately related to selection toward a decreased reliance on microbes, it raises the possibility that the proximate drivers include parallel changes in mechanisms mediating host-microbe interactions. Consistent with this possibility, both birds and bats have lost large numbers of genes involved in immune function, concomitant with a reduction in genome size (36, 37), including reduced interferon (IFN) locus copy number (38, 39). In bats, genes thought to be involved in minimization of DNA damage as a metabolic consequence of flight have been linked to immune function and are also under positive selection (36), suggesting that adaptation to flight may have affected key aspects of immunity. Continuous and dampened expression of certain immune genes has been proposed to allow bats to simultaneously manage flight-induced immune damage and to tolerate a large number of viral pathogens without experiencing disease (36, 40). Interestingly, our results suggest that bats also host significantly more potentially pathogenic bacteria than other mammals (Tukey’s *post hoc* test, *P* < 0.001), implying that links between flight and antiviral immunity may affect antibacterial immunity as well.

We cannot completely discount the possibility that the similarities we observed between bird and bat gut microbiomes are due at least in part to factors also correlated with flight. For example, birds and bats both excrete uric acid, in contrast to most other mammals. If uric acid or associated compounds interfere with DNA extraction or somehow compromised the microbial DNA prior to extraction, our observed sequence profiles might not accurately represent the relevant communities *in vivo*. However, nonavian reptiles, which also excrete uric acid, resemble nonflying mammals in their microbiome taxonomic compositions more than they resemble either birds or bats (Fig. 2). In addition, in birds, unlike bats, uric acid and feces mix directly in the cloaca before excretion. Finally, a subset of our bird samples was collected via intestinal aspiration, prior to the addition of uric acid in the cloaca, and analyses performed with this subset of samples did not differ substantially from those performed on feces (MRM fecal: *R*^2^ = 0.02, *P* = 0.001 for host phylogeny, *P* = 0.778 for diet; intestinal: *R*^2^ = 0.02, *P* = 0.001 for host phylogeny, *P* = 0.123 for diet). Consequently, this technical artifact explanation appears unlikely.

The observed bird-bat similarities may also be due to the captivity state of the source animals, in that much higher proportions of bird and bat samples were from wild individuals than those of nonbat mammals. However, we found that captivity only explains a small amount of the variation in our data (see Table S2), consistent with a previous study showing that the effects of captivity on the animal gut microbiome is not strong across mammals but rather varies markedly across host orders (22). Furthermore, a recent study of strictly wild animals also found higher levels of phylosymbiosis in mammals than in other vertebrate groups, including birds (17), consistent with captivity being an unlikely explanation for our observations.

10.1128/mBio.02901-19.3 TABLE S2 Adonis effect sizes. Download Table S2, DOCX file, 0.1 MB. Copyright © 2019 Song et al. 2019 Song et al. This content is distributed under the terms of the Creative Commons Attribution 4.0 International license.

Our data also suggest insectivory could be playing a role in microbiome specificity, at least in mammals. Although diet explained only a very small proportion of variance in bird microbiomes, and insectivorous mammals were not tightly clustered in our analysis (Fig. S3B), insectivorous mammals harbored some of the least mammalian-specific microbes (Fig. 4) and, like bats and birds, harbored higher proportions of *Proteobacteria* (Fig. S1). Most individual lineages of insectivores were not sampled deeply enough to accurately determine the strength of phylosymbiosis, but insect-eaters like armadillos (Cingulata) did have some of the weakest signals in our data set (Fig. 5). Previous work has reported some degree of convergence in the gut microbiomes of insectivorous mammals (20). Genomic analysis of insectivorous mammals also indicates convergent retention of functional chitinase genes expected to play a role in digesting the exoskeletons of their prey, potentially indicating a shift in reliance away from microbial and toward host function (41). If this is the case, the major shifts in host specificity we observe along independent branches on the vertebrate phylogeny in our data set could potentially be used to identify parallel changes in host genes mediating gut microbiome diversity.

Collectively, our results suggest a striking convergent loss of host-microbe associations with the evolution of powered flight, breaking a conserved and consistent pattern of phylosymbiosis observed in nonflighted mammals. We propose that this loss of microbiome specificity represents not just a passive shift in neutral exposure to microbial diversity but rather a fundamental and convergent shift in the physiological mechanisms responsible for maintaining host-specific gut microbiomes. Though these mechanisms are not yet well understood, the hypothesis of convergence between bats and birds offers novel opportunities for their discovery and validation via comparative approaches. Flight need not be the only mediator of such decreased reliance; indeed, the other mammalian taxa shown here to have relatively less-specific microbiomes offer additional opportunities for such comparisons. Our findings also raise a question not often voiced: what are the evolutionary and metabolic costs of maintaining a specific microbiome?

## MATERIALS AND METHODS

### Sample collection.

Through a large collaborative network of researchers and zoo directors, samples were collated from museum collections, zoos, reserves, and populations in their natural habitats (see Data Set S1 in the supplemental material). Collections were conducted under approved IACUC protocols, and appropriate permits were obtained for sample collection and export where necessary. Sampling of fecal material from animals in zoos and reserves involved collecting up to 2 g of fresh fecal material using sterile swabs (BD CultureSwab or equivalent) within minutes to hours of deposition, which was immediately frozen, and stored at −20°C until DNA extraction. Samples from museum collections included fecal or intestinal contents subsampled from frozen specimens using sterile implements or swabs. The 214 Smithsonian specimens were aspirations of large intestine (colon) from freshly killed wild birds. Samples taken directly from wild populations included fecal contents stored in RNAlater, on Whatman FTA cards, or in 95% ethanol (EtOH) (Data Set S1).

### Sequencing.

Samples collected for this project were processed using the Earth Microbiome Project standard processing protocols (19). A subset of samples originally collected for previously published studies (2, 3, 20) were reprocessed using these protocols. Briefly, sterile cotton-tipped swabs were used to transfer approximately 50 mg of sample to 96-well PowerSoil PowerMag DNA extraction plates (Qiagen), which were homogenized using a TissueLyser beadbeater (Qiagen); extractions were completed on a KingFisher magnetic bead transfer robot (Thermo Fisher). From eluted DNA, triplicate PCRs using the 515f/806r EMP primers amplified the V4 region of the 16S rRNA gene, and pooled amplicons were sequenced on Illumina MiSeq and HiSeq instruments.

### Data processing and metadata curation.

To ensure that data from new samples were processed consistently with samples from prior studies, we uploaded all new sequence data to the Qiita web-based microbiome analysis platform for initial processing (42). All sequences were demultiplexed and quality filtered using Qiita defaults, and forward reads were trimmed to 100 bp prior to processing with Deblur to remove sequencing errors (43). To avoid potential artifactual sequences, we used the “positive-filtered” output table from Deblur, which retains only those sequences that approximately match the Greengenes 13_8 16S reference database (44).

Deblurred ASV tables and sample metadata were downloaded from each study and further processed using Qiime2 (45). Per-study ASV tables were combined, and a phylogeny was estimated with SEPP (46) as implemented in the q2-fragment-insertion Qiime2 plugin (47). Taxonomy was assigned using the Qiime2 naive Bayes feature classifier trained against the Greengenes 13_8 reference (44). ASVs classified as from mitochondria or chloroplasts were excluded from further analysis. Samples were rarefied to 10,000 reads. To prevent large imbalances in sample number among species from influencing ordinations, we randomly subsampled the rarefied data set to a maximum of 5 individuals per species, and these tables were used throughout the analysis.

Metadata tables for all samples were combined, and host-level metadata were added from several sources. To accomplish this, we first curated host species names and taxonomies against the NCBI taxonomy database using the Taxon Names Resolver python package (48). Samples for which the provided species name could not be automatically resolved were manually curated, as were taxa for which individual taxonomic levels were missing from NCBI taxonomy (e.g., Cetartiodactyla, which is an unranked level in NCBI). Curated species names were then matched against the EltonTraits ecological trait database, and any missing taxa checked and manually curated if necessary (49). Metadata on bird migration were compiled by K.W. from sources cited in Data Set S1.

To obtain an approximate host phylogeny, we matched curated species names to the TimeTree database (24). Unmatched taxa were manually curated and matched to the corresponding species binomial in the TimeTree database where possible. For taxa not present in the TimeTree database, we substituted a close congeneric species if (i) one was present in the TimeTree database, and (ii) there were no other congeneric species present in our sample set.

### Beta diversity analyses.

Beta diversity measures were calculated for the complete data set in Qiime2, using the SEPP insertion phylogeny for UniFrac phylogenetic metrics (50). Principal-coordinate analyses were performed in Qiime2 and visualized using Emperor (51) and ggplot2 (52) in R (53). Tests for categorical differences in beta diversity were performed using PERMANOVA (54) as implemented in R’s vegan package (55). To compare differences in beta diversity to differences in host diet and phylogeny, we used multiple regression of matrices as implemented in the ecodist package in R (56). To represent host evolutionary distance in these regressions, we used patristic distances derived from the TimeTree host phylogeny. To represent dietary dissimilarities, we used Bray-Curtis distances derived from the EltonTraits quantitative dietary compositions. To assess how correlations between microbiome dissimilarity and host phylogenetic and dietary dissimilarities were sensitive to microbial phylogenetic resolution, we used the SEPP insertion tree as an estimate of microbial phylogeny and compared correlations at different bacterial phylogenetic resolutions to the above host dietary and phylogenetic distances using Mantel tests in the beta diversity through time algorithm (5).

### Alpha diversity analyses.

Alpha diversity measures (observed OTUs, Faith’s phylogenetic diversity, and Shannon diversity) were calculated from the ASV tables rarefied to 10,000 sequences per sample in Qiime2. The SEPP insertion tree was used as an estimate of the bacterial phylogeny for the phylogenetic diversity measure. Differences in alpha diversity by categorical metadata variables were tested using ANOVA in R, with pairwise differences between categories assessed with the multcomp package (57). Differences in alpha diversity by continuous metadata variables were assessed using linear regressions.

### Host specificity analyses.

We used several methods to assess host specificity in microbiomes. One measure of specificity is phylosymbiosis, or the correlation between host phylogenetic distance and microbiome dissimilarity. To quantify phylosymbiosis systematically across taxa, we implemented a version of the Mantel test as used by Nishida and Ochman (12), comparing measures of microbial beta diversity to host patristic distances derived from the TimeTree time-calibrated phylogeny. We used the Jaccard dissimilarity metric for most analyses, as this measure reflects the proportion of shared exact ASVs, and so is more sensitive to recent microbial codiversification than UniFrac (10). To prevent the possibility of zero-length within-species branches from biasing regressions, we randomly subsampled the data set to a single representative per species (25); for visualization purposes (e.g., Fig. 5), we plotted all between-species points while excluding within-species points. We calculated the Mantel Pearson correlation between microbiome dissimilarity and host phylogenetic distance and the partial Mantel correlation (conditioned on dietary distances) at every node of the host phylogeny that contained at least seven tips using the EcoPy package (58). We visualized the strength of the correlation across the host phylogeny using the iToL web-based tree rendering tool (59) (Fig. 1).

We also tested the specificity of ASVs to particular host taxa using an implementation of the environmental entropy calculation in reference 19. Briefly, we calculated the host taxonomic distribution of each ASV, represented by a vector of integers corresponding to the number of samples from each host taxon in which the ASV was observed. We only considered ASVs observed in at least ten samples. We transformed these integer vectors to per-ASV proportional values and visually illustrated host taxonomic distributions for ASVs with stacked bar charts (Fig. 3). We quantified specificity of these host taxonomic distributions using Pielous’s evenness statistic: maximally specific ASVs are found in just one host taxon, while minimally specific ASVs are found evenly across all host taxa. Because our sample set was not perfectly balanced across host taxa, biases in sampling could make ASV distribution appear uneven simply due to uneven sampling. Thus, we calculated a standardized effect size (SES) for this statistic. We permuted sample assignment within each ASV in the observation table, such that each ASV was found in the same number of samples in the permuted table as in the raw table. We then calculated the SES as the raw evenness statistic for an ASV, minus the mean of 100 permuted statistics, divided by the standard deviation of the permuted statistics. We calculated a separate Pielou’s evenness SES value for each ASV based on its distribution across mammalian order-level taxa and across avian order-level taxa.

To assess the degree to which individual ASVs were specific to mammalian or avian taxa, we calculated their likelihood of being observed in mammalian or avian samples, respectively. To do this, we used log risk ratios, or the log of the ratio of an ASV’s prevalence in mammalian samples to its prevalence in avian samples. Because this is undefined for ASVs never found in either mammals or birds, we set values for these taxa to ±3, which just exceeded the range of defined values in our data set. We then calculated an average per sample as a measure of how specific ASVs in each sample were to mammals or birds.

### Microbial characteristics.

To predict high level microbial phenotypic characteristics using 16S sequence data, we implemented BugBase (https://bugbase.cs.umn.edu/) (30). We first clustered the deblurred ASVs against the Greengenes reference database (v13_5) at 99% identity and then rarefied the data to 5,000 sequences per sample.

To rank microbial phyla based on their association with birds, we used a regression approach for detecting differential abundances in microbiome data (60). ASVs were first collapsed at the phylum level based on taxonomy assigned using the Qiime2 naive Bayes feature classifier trained against the Greengenes 13_8 reference as described above. A model was built testing for differences among host classes, with Mammalia serving as the reference, using a batch size of 10 and an epoch of 1,000,000.

### Data availability.

Sequence data and metadata tables are available without restriction in Qiita (https://qiita.ucsd.edu/study/description/11166; full list of study identifiers [IDs] is in Data Set S1) and EBI (accession no. PRJEB35449). Analysis notebooks are available on Github (https://github.com/tanaes/tetrapod_microbiome_analysis).
