# Supplementary figures and images for: Comparative Analyses of Vertebrate Gut Microbiomes Reveal Convergence between Birds and Bats
Source: mBio. 2020 Jan 7;11(1):e02901-19. doi: 10.1128/mBio.02901-19 (PMC6946802; doi:10.1128/mBio.02901-19)

A)

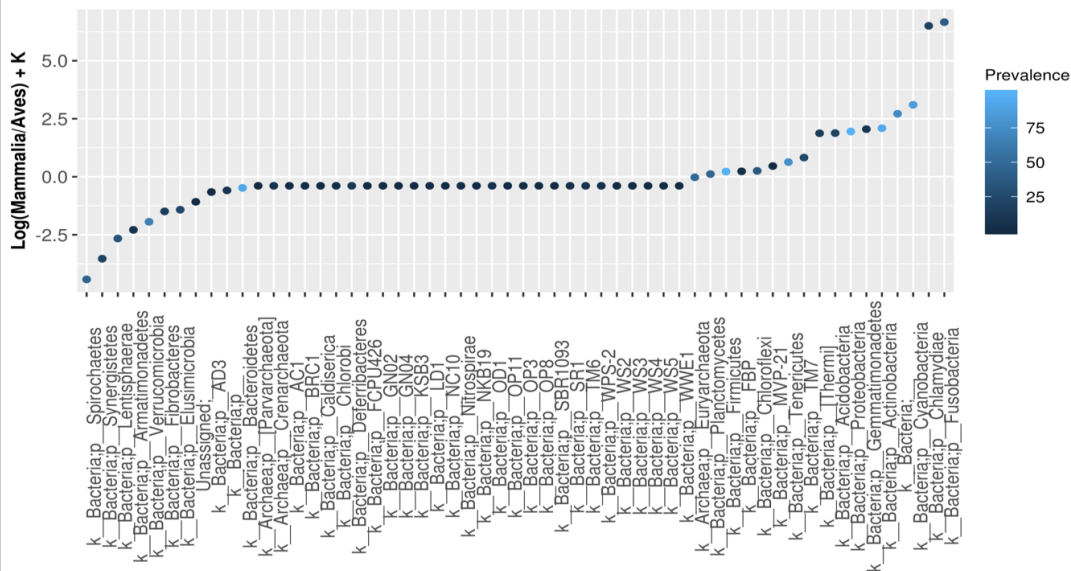

B)

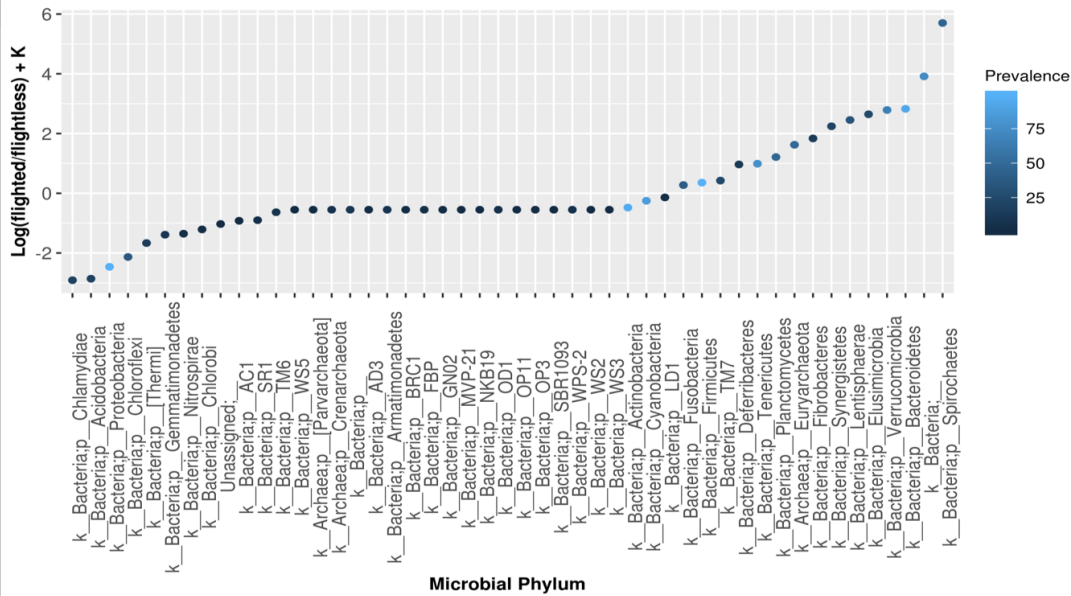

Microbial Phylum

C)

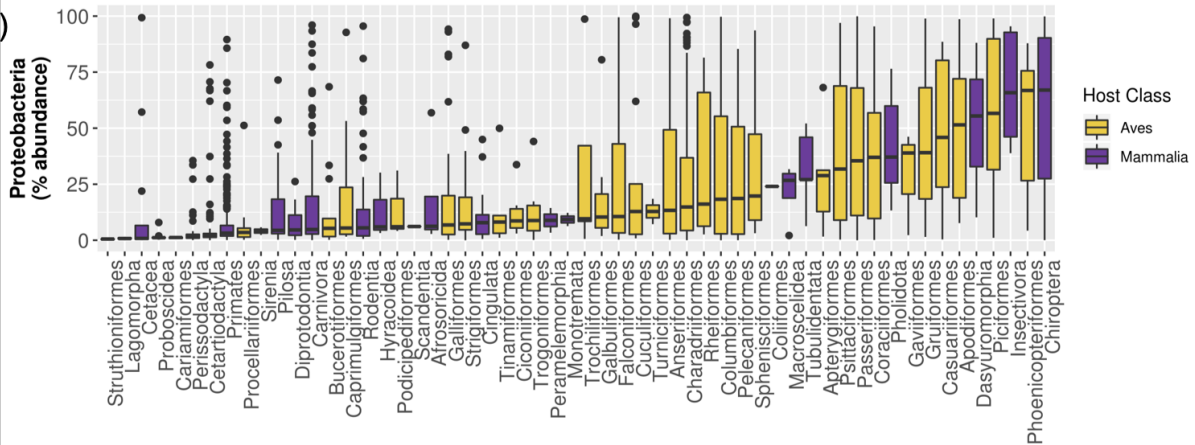

D)

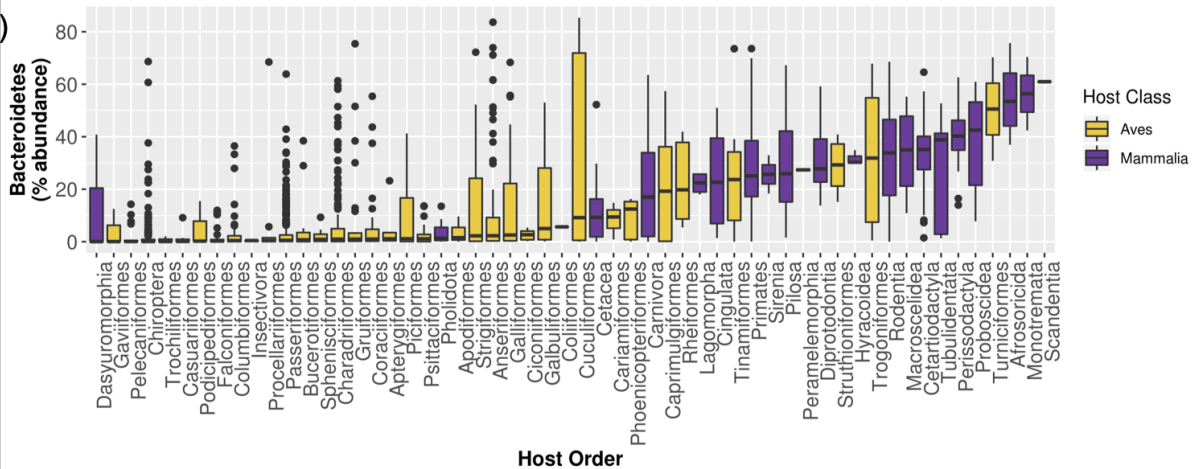

Host Order

Supplement: FIG S1 [file mBio.02901-19-sf001.pdf]

A) Unweighted UniFrac

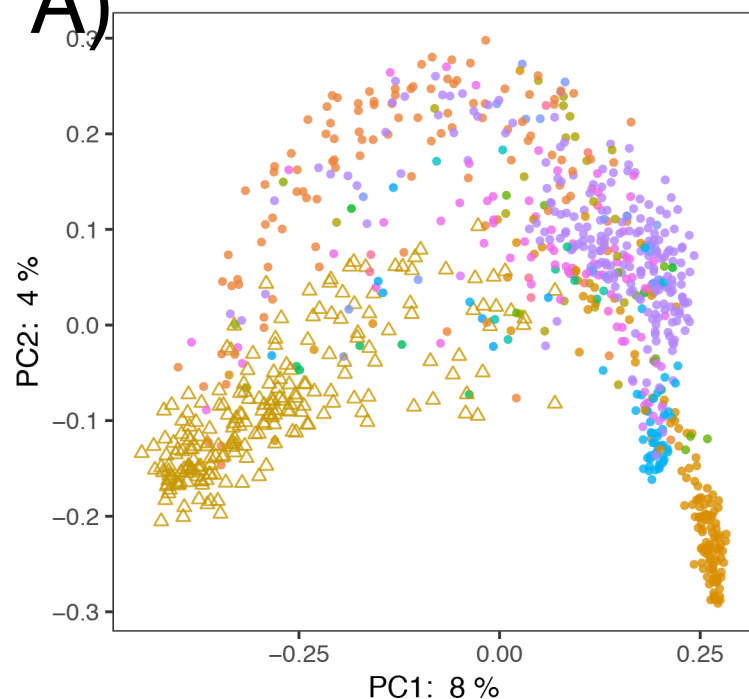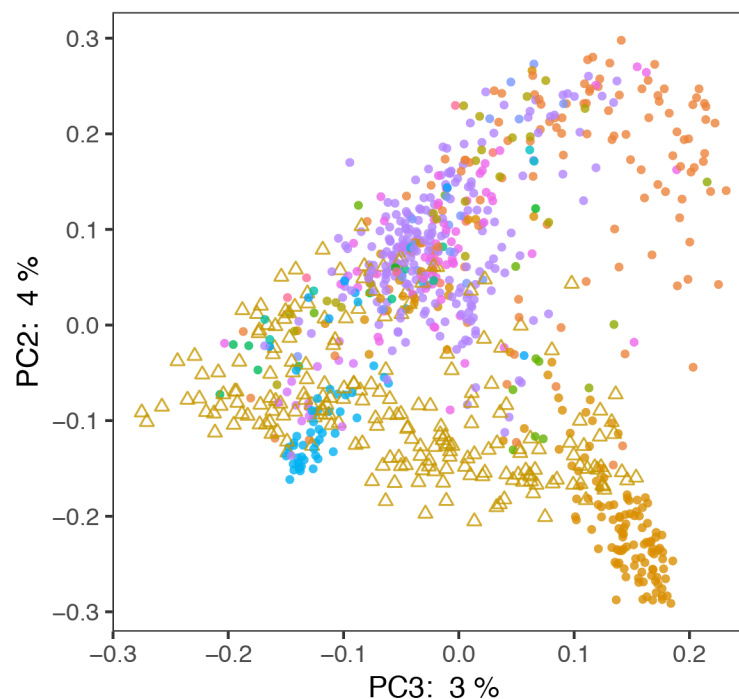

Taxonomy\_Order

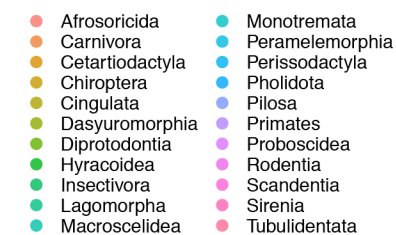

is\_bat

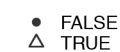

B) Unweighted UniFrac

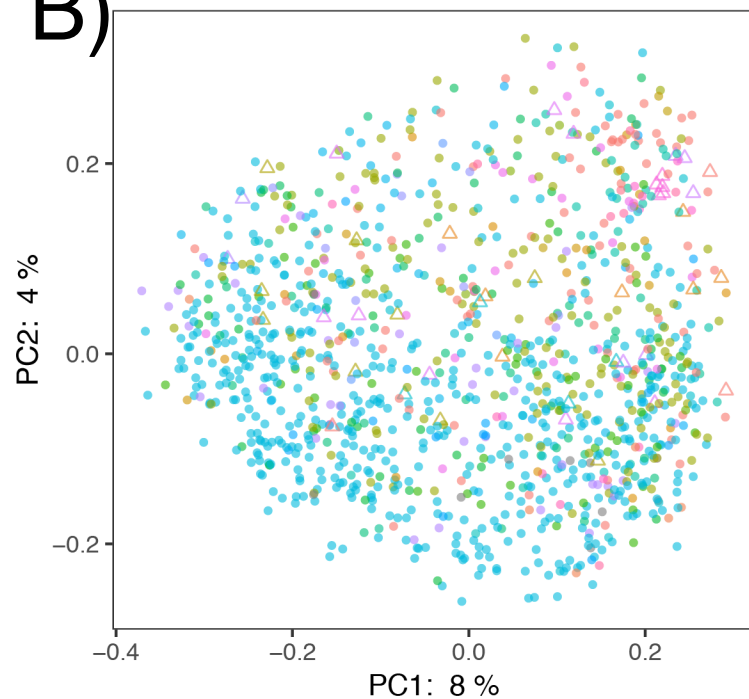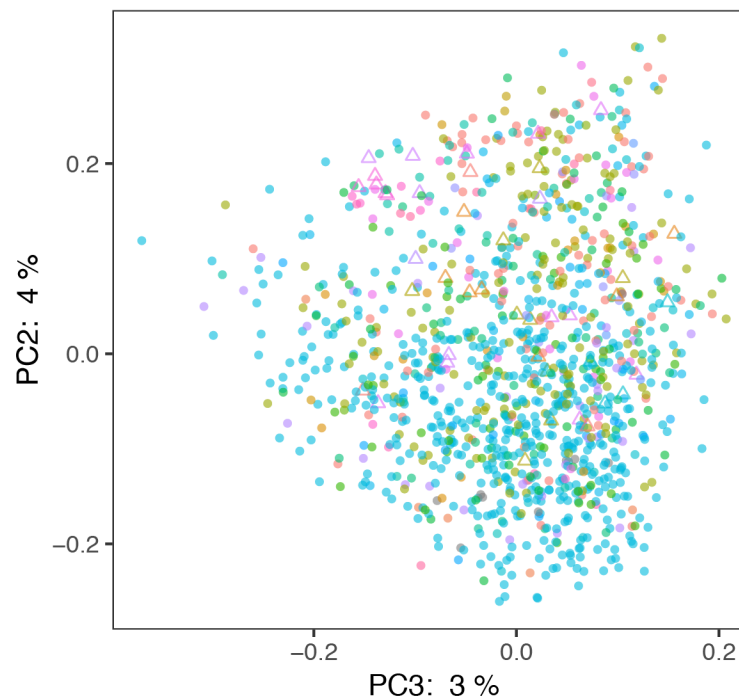

flight\_status

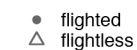

Taxonomy\_Order

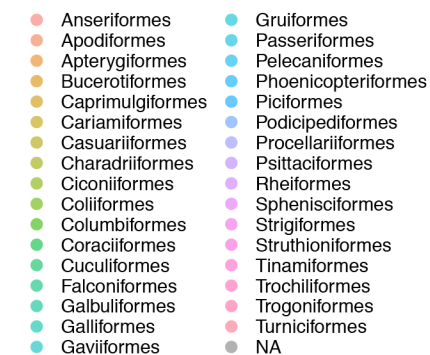

Supplement: FIG S2 [file mBio.02901-19-sf002.pdf]

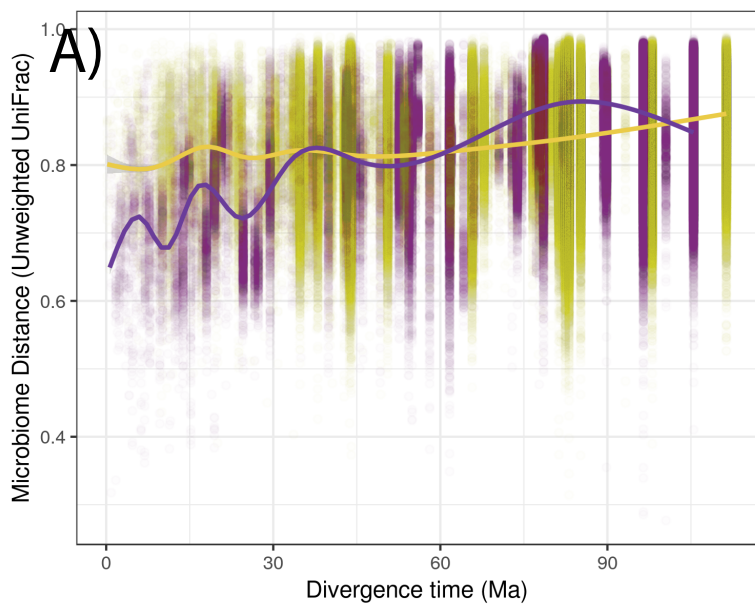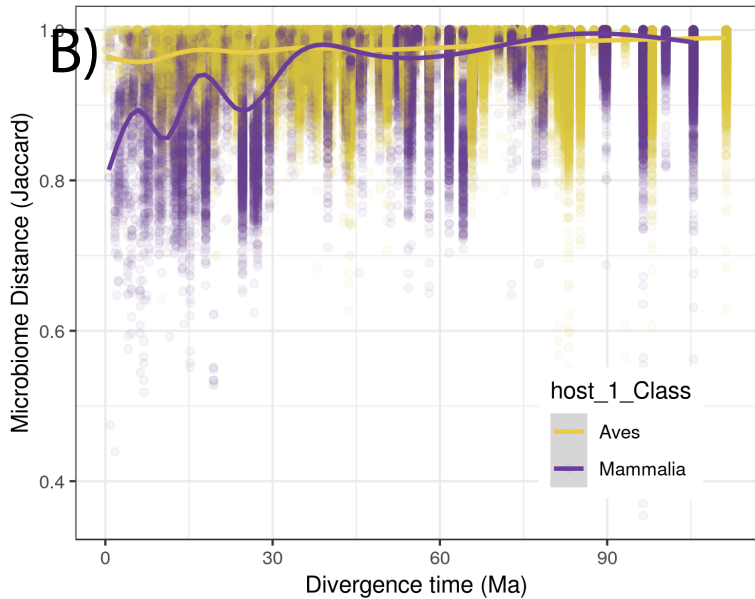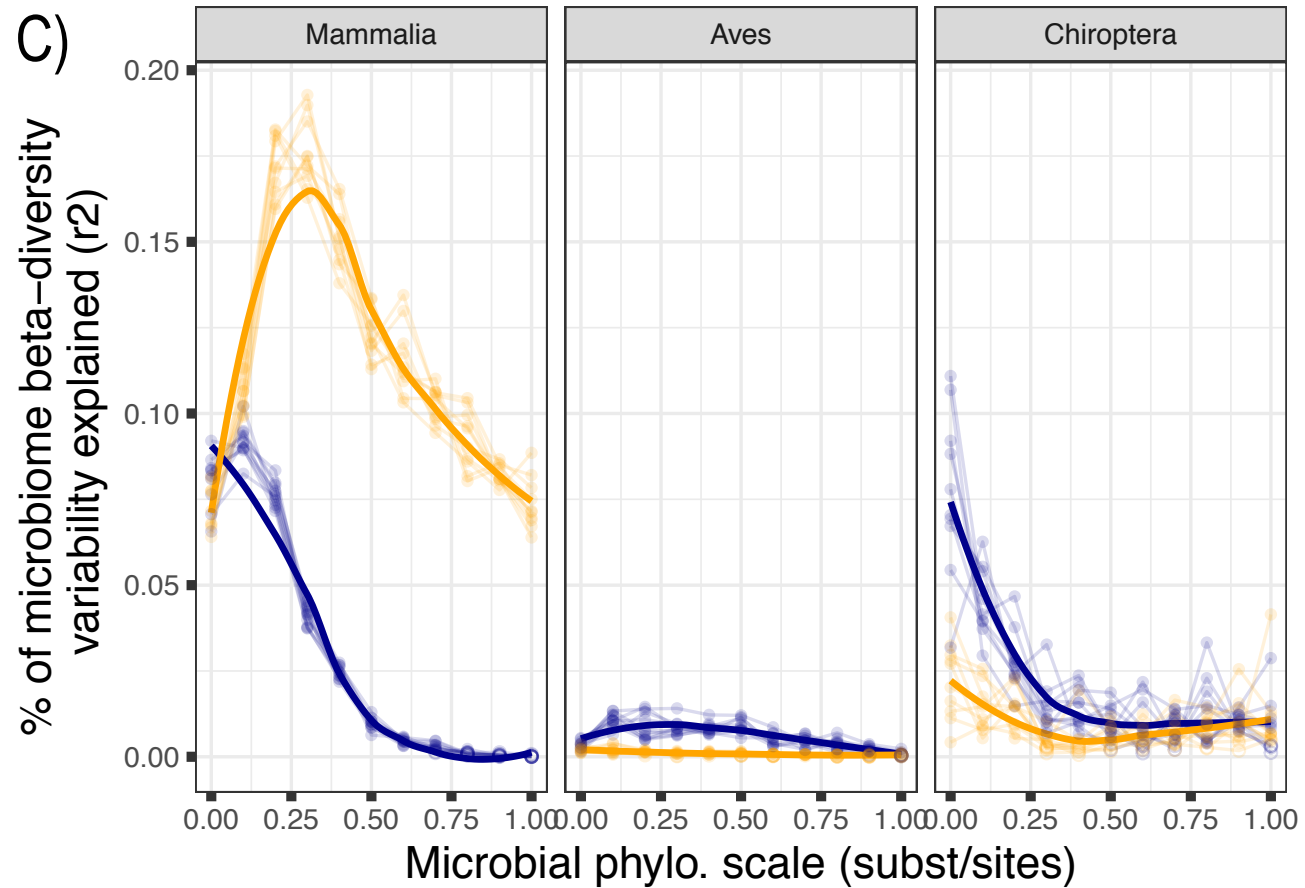

Supplement: FIG S4 [file mBio.02901-19-sf004.pdf]

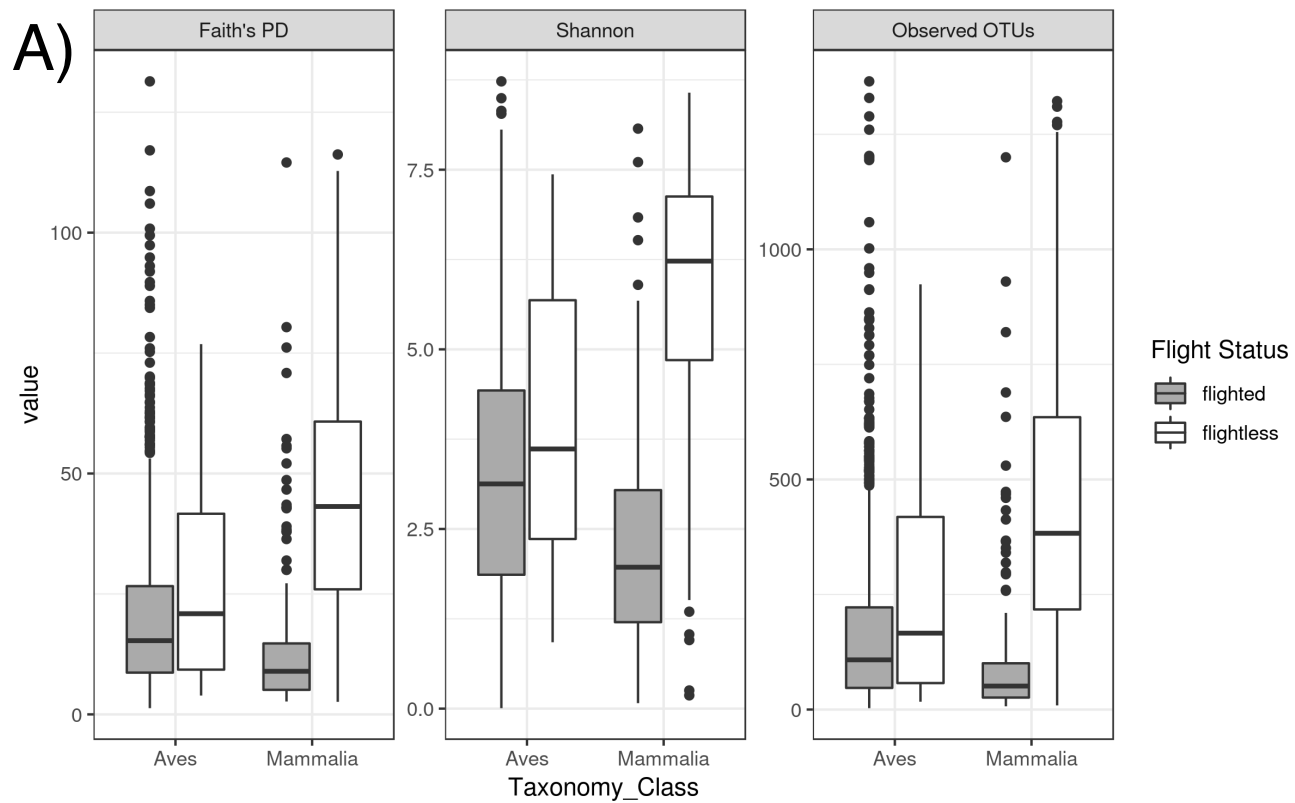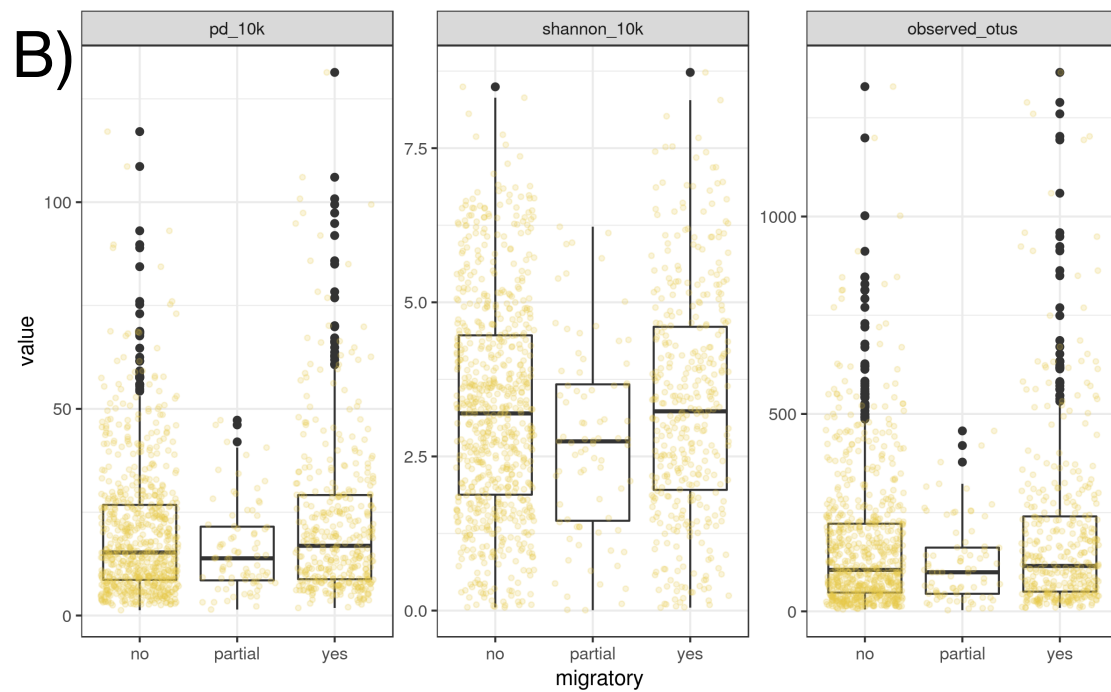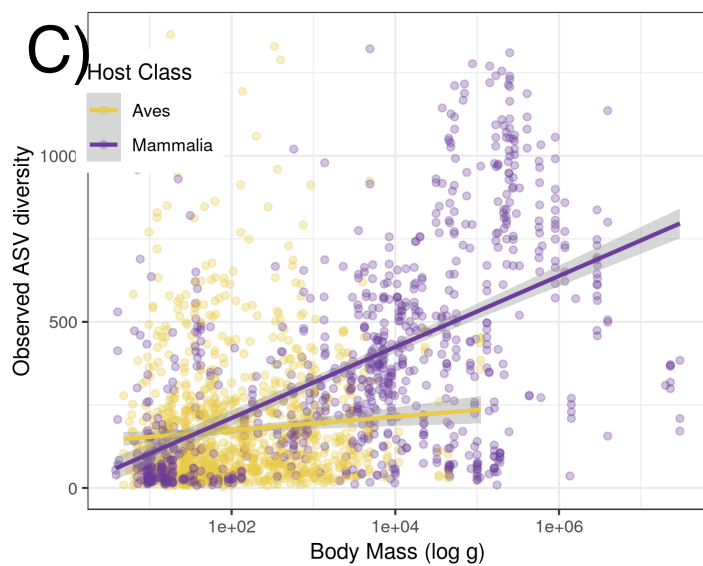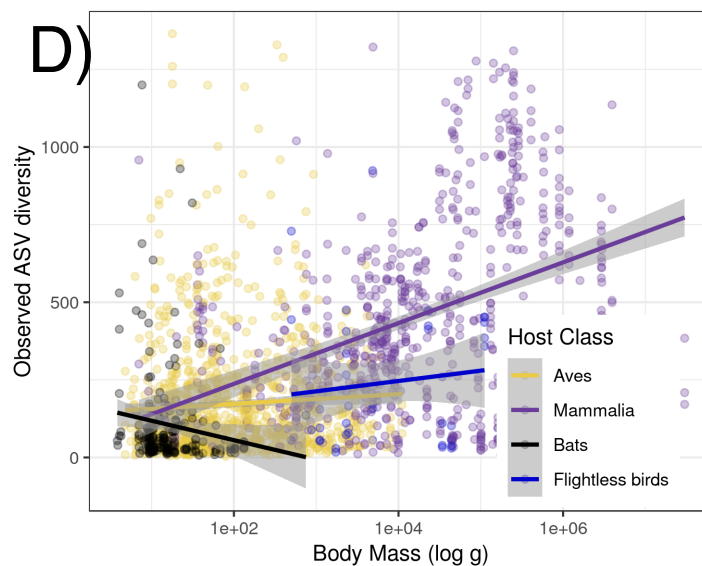

Supplement: FIG S5 [file mBio.02901-19-sf005.pdf]

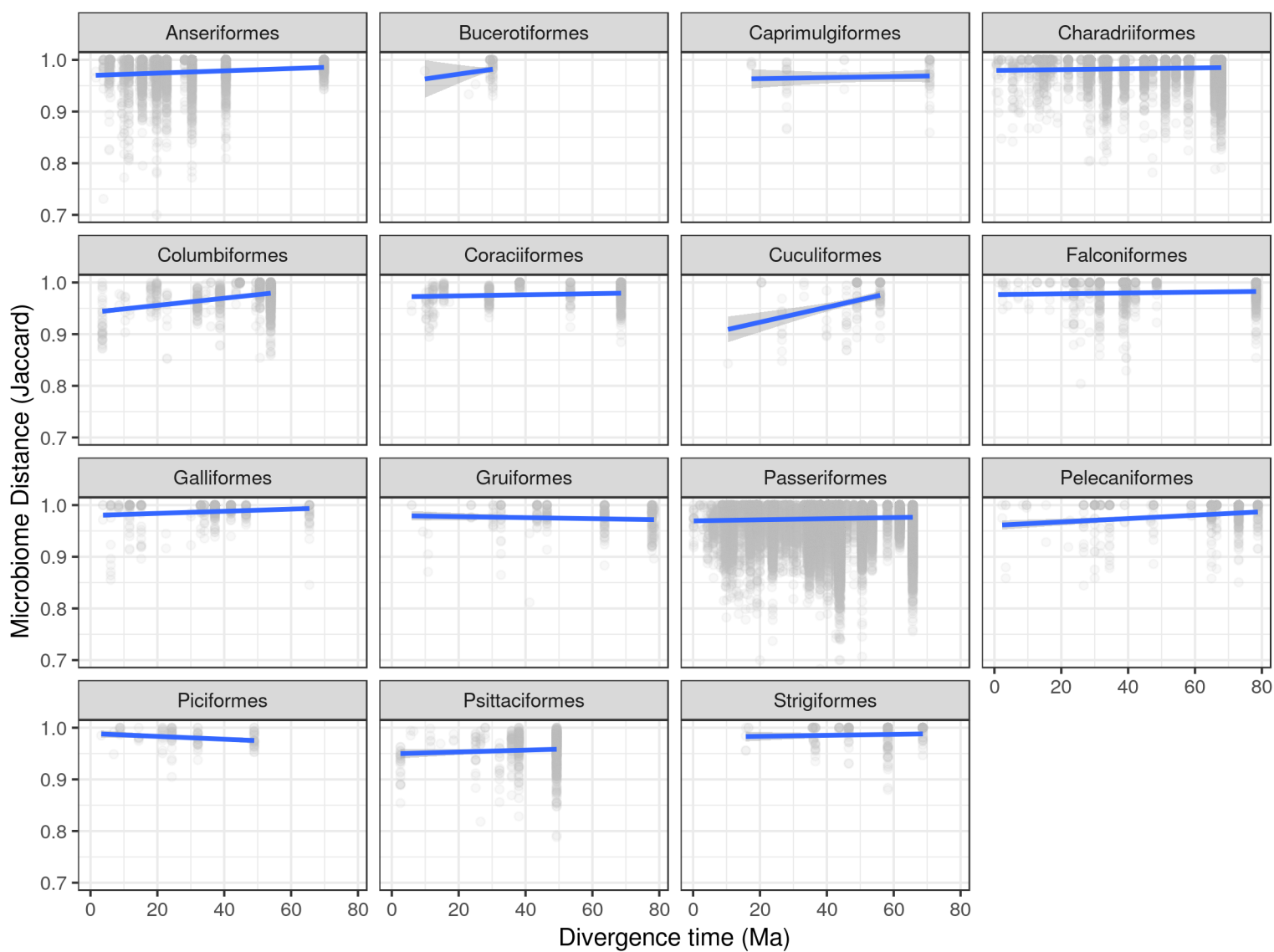

Supplement: FIG S7 [file mBio.02901-19-sf007.pdf]

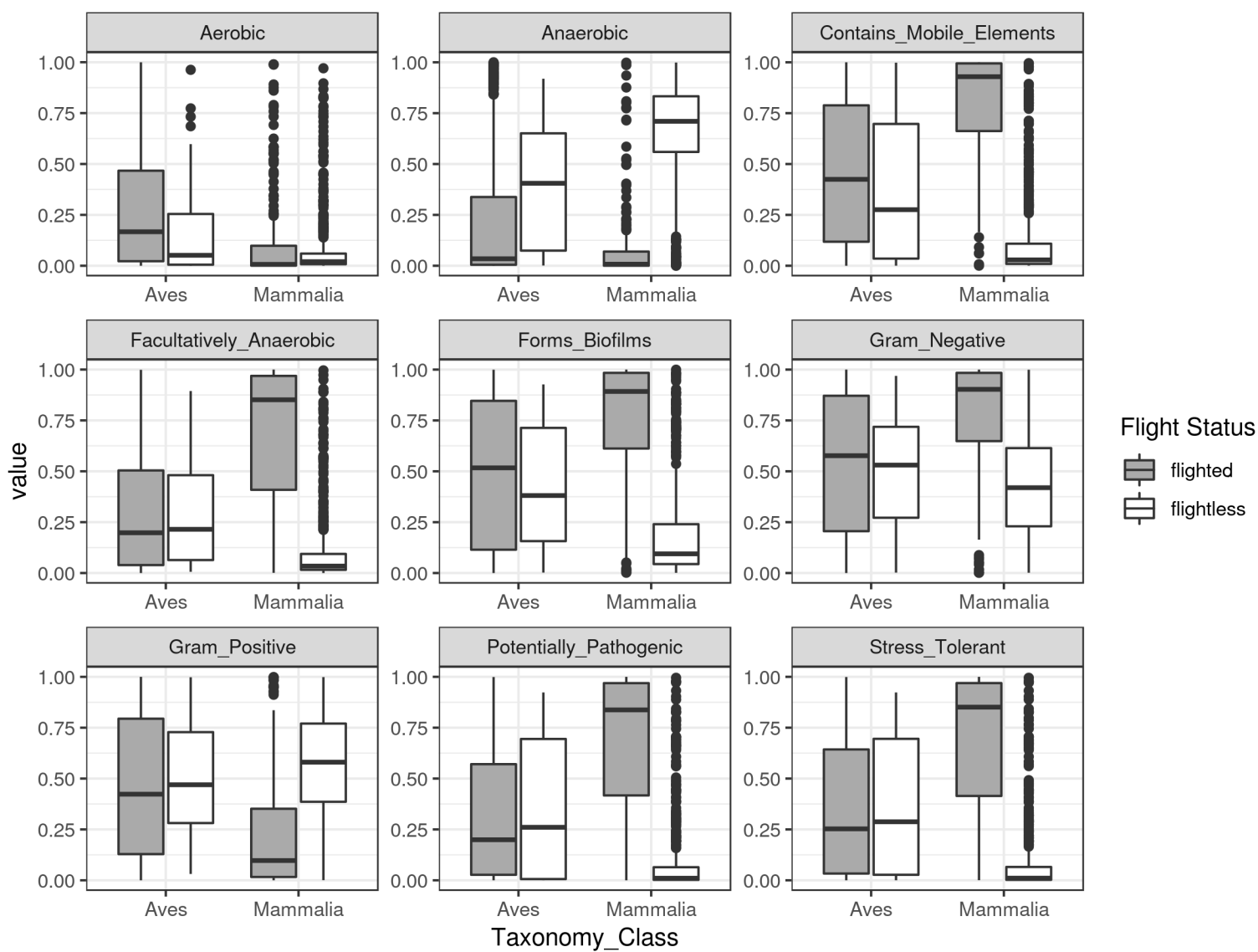

Supplement: FIG S6 [file mBio.02901-19-sf006.pdf]
