# Supplementary material for: Comparative Analyses of Vertebrate Gut Microbiomes Reveal Convergence between Birds and Bats
Source: mBio. 2020 Jan 7;11(1):e02901-19. doi: 10.1128/mBio.02901-19 (PMC6946802; doi:10.1128/mBio.02901-19)
Supplement: TABLE S1 [file mBio.02901-19-st001.docx]

**Table S1. MRM regression effect sizes of diet and phylogenetic distance**

|  | **R^2^_model_** | ***p*_phylogeny_** | ***p*_diet_** |
| --- | --- | --- | --- |
| Mammals | 0.173 | 0.001 | 0.001 |
| Birds | 0.017 | 0.001 | 0.415 |
| Reptiles* | 0.035 | 0.003 | – |
| Amphibians* | 0.030 | 0.021 | – |

* reptile and amphibian samples lack quantitative diet metadata, and only regressed against phylogenetic distance
