# Supplementary material for: Comparative Analyses of Vertebrate Gut Microbiomes Reveal Convergence between Birds and Bats
Source: mBio. 2020 Jan 7;11(1):e02901-19. doi: 10.1128/mBio.02901-19 (PMC6946802; doi:10.1128/mBio.02901-19)
Supplement: FIG S3 [file mBio.02901-19-sf003.pdf]

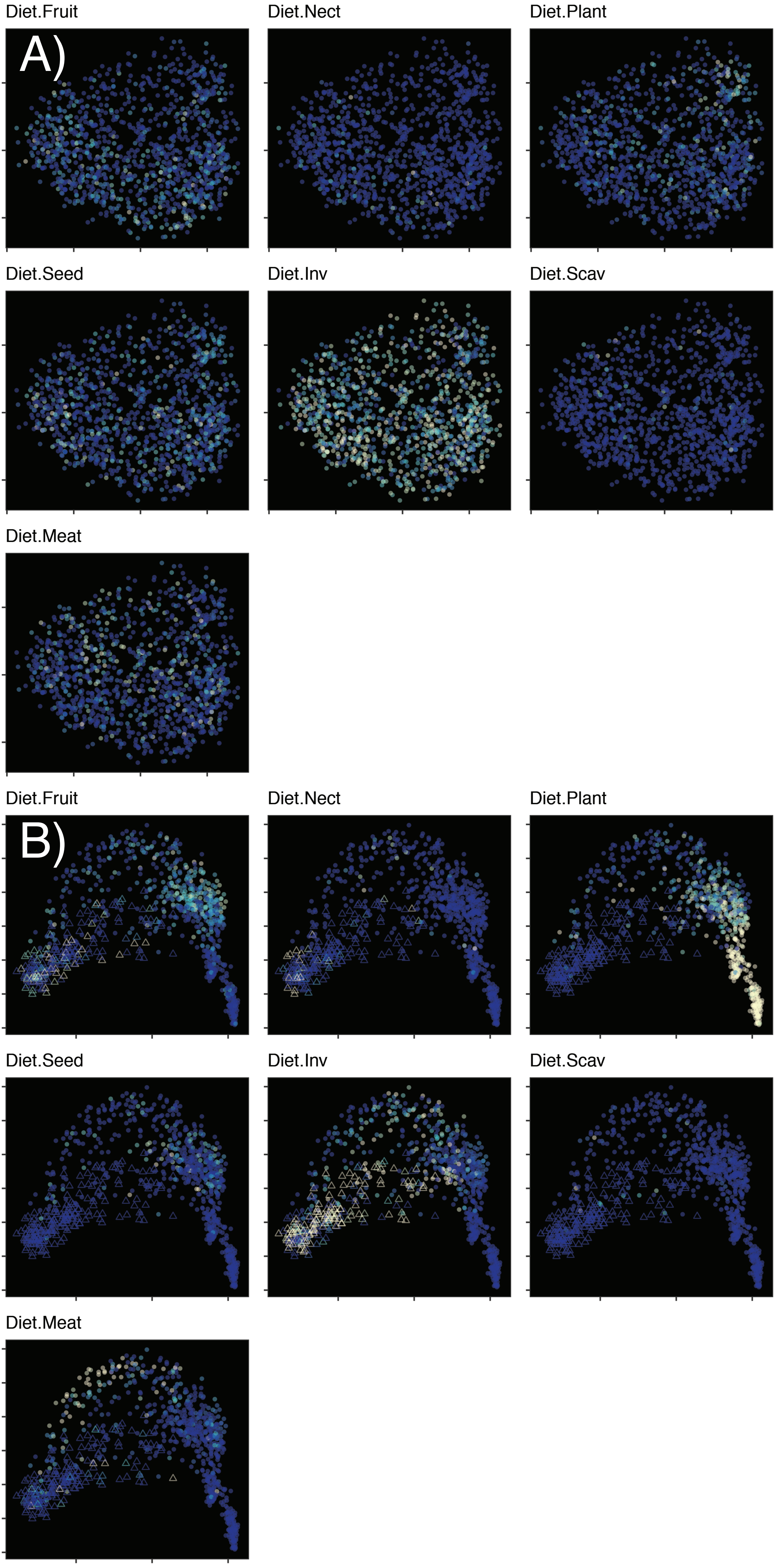
A)
Diet.Fruit
Diet.Nect
Diet.Plant
Diet.Seed
Diet.Inv
Diet.Scav
Diet.Meat
B)
Diet.Fruit
Diet.Nect
Diet.Plant
Diet.Seed
Diet.Inv
Diet.Scav
Diet.Meat
