# Supplementary material for: Comparative Analyses of Vertebrate Gut Microbiomes Reveal Convergence between Birds and Bats
Source: mBio. 2020 Jan 7;11(1):e02901-19. doi: 10.1128/mBio.02901-19 (PMC6946802; doi:10.1128/mBio.02901-19)
Supplement: TABLE S2 [file mBio.02901-19-st002.docx]

**Table S2. Adonis effect sizes.**

|  | **R^2^_all_** | **R^2^_birds_** | **R^2^_mammals_** |
| --- | --- | --- | --- |
| Taxonomy (class) | 0.049 | – | – |
| Taxonomy (order) | – | 0.071 | 0.207 |
| Flight | 0.027 | 0.003 | 0.076 |
| Captivity* | 0.010 | 0.005 | 0.005 |
| Sample Type* | 0.014 | 0.018 | 0.010 |
| Preservative* | 0.046 | 0.030 | 0.031 |

All terms have a p-value of 0.001.

* Captivity, Sample Type, and Preservative effect sizes after regression on Taxonomy
